# Characterization of Chlorhexidine-Loaded Calcium-Hydroxide Microparticles as a Potential Dental Pulp-Capping Material

**DOI:** 10.3390/bioengineering4030059

**Published:** 2017-06-22

**Authors:** Balasankar M. Priyadarshini, Subramanian T. Selvan, Karthikeyan Narayanan, Amr S. Fawzy

**Affiliations:** 1 Discipline of Oral Sciences, Faculty of Dentistry, National University of Singapore, 11 Lower Kent Ridge Road, Singapore 119083, Singapore; a0106278@u.nus.edu; 2 Department of Chemistry, Myongji University, Natural Science Campus, 116 Myongji-ro, Cheoin-gu, Yongin, Gyeonggi-do 449-728, Korea; selvant@mju.ac.kr; 3 Institute of Bioengineering and Nanotechnology, 31 Biopolis Way, The Nanos, #04-01, Singapore 138669, Singapore; karthisurya2002@gmail.com

**Keywords:** chlorhexidine, dentin surfaces, dentinal tubules, microparticles, calcium hydroxide

## Abstract

This study explores the delivery of novel calcium hydroxide [Ca(OH)_2_] microparticles loaded with chlorhexidine (CHX) for potential dental therapeutic and preventive applications. Herein, we introduce a new approach for drug-delivery to deep dentin-surfaces in the form of drug-loaded microparticles. Unloaded Ca(OH)_2_ [Ca(OH)_2_/Blank] and CHX-loaded/Ca(OH)_2_ microparticles were fabricated by aqueous chemical-precipitation technique. The synthesized-microparticles were characterized in vitro for determination of surface-morphology, crystalline-features and thermal-properties examined by energy-dispersive X-ray scanning and transmission electron-microscopy (EDX-SEM/TEM), Fourier-transform infrared-spectroscopy (FTIR), X-ray diffraction (XRD), thermogravimetric analysis (TGA) and differential scanning-calorimetry (DSC). Time-related pH changes, initial antibacterial/biofilm-abilities and cytotoxicity of CHX-loaded/Ca(OH)_2_ microparticles were evaluated. Microparticles were delivered to dentin-surfaces with subsequent SEM examination of treated dentin-substrates. The in vitro and ex vivo CHX-release profiles were characterized. Ca(OH)_2_/Blank were hexagonal-shaped with highest *z*-average diameter whereas CHX-inclusion evidenced micro-metric spheres with distinguishable surface “rounded deposits” and a negative-shift in diameter. CHX:Ca(OH)_2_/50 mg exhibited maximum encapsulation-efficiency with good antibacterial and cytocompatible properties. SEM examination revealed an intact layer of microparticles on exposed dentin-surfaces with retention of spherical shape and smooth texture. Microparticles loaded on dentin-surfaces showed prolonged release of CHX indicating substantial retention on dentin-substrates. This study validated the inherent-applicability of this novel drug-delivery approach to dentin-surfaces using micro-metric CHX-loaded/Ca(OH)_2_ microparticles.

## 1. Introduction

Restoration of tooth structure with retention of pulp vitality is an important essence of restorative dentistry [1]. Vital pulp therapy (VPT) provides the advantage of preserving the dental pulp tissue that has been compromised by caries, trauma or restorative procedures and stimulates the remaining pulp to regenerate reparative dentin [1,2]. Clinical studies have highlighted the success of materials such as calcium hydroxide [Ca(OH)_2_], zinc oxide eugenol (ZOE) and mineral trioxide aggregate (MTA) in effective pulp-capping procedures [3,4]. Of these, calcium hydroxide was originally introduced to the field of endodontics as an intracanal pulp-capping/apexification agent owing to its high alkalinity and biocompatible nature; which however has extensive applications in modern clinical dentistry [5]. Most studies on the applications of calcium hydroxide have focused on some of its biological properties such as antibacterial efficacy, dentin-remineralization and stimulation of reparative bridge formation [6,7]. Traditionally, Ca(OH)_2_ dressings (such as the commercially available products; Dycal^®^ DENTSPLY and Pulpdent^®^ Paste) have been applied on the deepest layer of the affected dentin intentionally left remaining over the dental pulp tissue. In a large percentage of cases, this aided in the formation of a calcified reparative barrier that prevents possible pulp exposure thereby protecting the underlying pulp tissue from further injury [8,9]. Moreover, the alkaline pH of Ca(OH)_2_ has been implied in the stimulation of reparative dentin formation [10]. Although benefits of Ca(OH)_2_ have been regarded, concerns such as pulp inflammation upon Ca(OH)_2_ exposure and reduced antibacterial activity due to formation of a defective barrier were previously addressed [11,12].

Chlorhexidine (CHX), a commonly-used dental antiseptic, has displayed a valuable therapeutic role as a pulp-capping disinfectant and non-specific Matrix Metallo Proteinases (MMPs) inhibitor with proven efficacy against MMP-2, -8, -9 trapped in dentin [13,14]. Often times, researchers have combined Ca(OH)_2_ with Chlorhexidine, by physical modification of both their commercially available forms to obtain pastes/powder for enhanced antimicrobial action and to achieve some degree of synergism [15,16]. Studies revealed that this combination effectively eradicated microorganisms that were not eliminated by Ca(OH)_2_ alone [16,17]. However, no additional advantage was reported [18]. Moreover, only few studies have perceived this combination for use in drug-delivery and scrutinized their resultant properties in vitro [19]. In this work, we have introduced a new approach to assess the potential of biodegradable drug-loaded microparticle formulation that can be used as an effective pulp-capping material with promising remineralization, MMPs inhibition and antibacterial properties. Chlorhexidine-loaded Ca(OH)_2_ [CHX-loaded/Ca(OH)_2_] microparticles were utilized for this purpose by virtue of the proven success of this combination [20]. Our proposed principle was to deliver the active-drug (CHX) to the exposed deepest dentin adjoining the pulpo-dentinal junction in the form of micron-sized particles for effective delivery, retention, and extended CHX-release over time. Upon dentin application, Ca(OH)_2_ microparticles eventually degrade slowly to release significant CHX-doses in a controlled manner at pre-determined time-periods. The general aim of this study was to explore the delivery of novel calcium hydroxide microparticles loaded with chlorhexidine for potential dental therapeutic and preventive applications. Accordingly, the following specific aims will be identified: to extensively characterize the synthesized CHX-loaded/Ca(OH)_2_ microparticles in vitro, in terms of particle-size, surface-charge and morphology, CHX-loading and entrapment, in vitro release profile, spectral analyses, pH measurement, cytotoxicity testing and antibacterial/biofilm activity. In this work, we have evaluated the in vitro properties of the synthesized microparticles in comparison to reference pulp capping materials: Dycal^®^ (Dentsply) and commercial Ca(OH)_2_ microparticles.

## 2. Materials and Methods

### 2.1. Reagents and Materials

Calcium chloride dihydrate (CaCl_2_·2H_2_O) (Mw~147.01 g/mol), sodium hydroxide (NaOH), 2% chlorhexidine gluconate, phosphate buffered saline (PBS), triton X-100 [C_14_H_22_O(C_2_H_4_O)_10_] and commercial Ca(OH)_2_ powder were obtained from Sigma-Aldrich (St. Louis, MO, USA). Brain heart infusion (BHI) broth and agar were obtained from Thermo-Scientific (Oxoid Limited, Hampshire, UK). Micro-brush applicators were purchased from 3M ESPE (St. Paul, MN, USA). Dycal^®^ was purchased from Dentsply (Caulk, Milford, DE, USA); the catalyst and base pastes of Dycal were mixed in the ratio of 1:1 according to manufacturer’s instructions. Whatman PTFE Membrane Filters (pore size: 0.45 μm; L × W: 300 mm × 640 mm) were purchased from Sigma-Aldrich (St. Louis, MO, USA). All chemicals used in this study, were of analytical grade and used without further purification.

### 2.2. Synthesis of Microparticles

**Figure 1:**
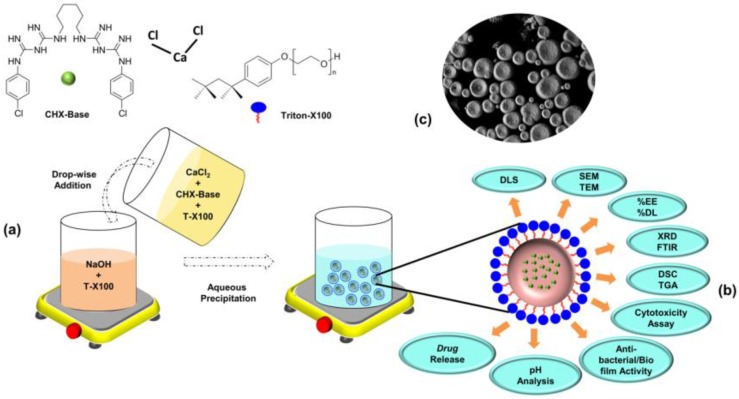
(**a**) Schematic illustration showing the synthesis and recovery of CHX-loaded/Ca(OH)_2_ microparticles by modified aqueous chemical-precipitation method. The aqueous solutions containing 0.3 mol/L of CaCl_2_·2H_2_O and 0.6 mol/L of NaOH were prepared separately with the supplementation of 0.15% of Triton X-100 to both the above formulated initial solutions. Different amounts of 25 and 50 mg of chlorhexidine (CHX) was added to the prepared CaCl_2_·2H_2_O solution, blended well and added drop-wise into the aqueous NaOH solution at about 50 °C. Two distinct phases consisting of a limpid supernatant and white chalky precipitate was observed at about 1 h. The supernatant was discarded and the precipitate was purified by centrifugation, vacuum desiccated to minimize the extent of carbonation process followed by storage at 4 °C for further investigation and characterization. Unloaded Ca(OH)_2_ microparticles (Ca(OH)_2_/Blank) were synthesized following the same method as mentioned above without the supplementation of CHX. (**b**) Following synthesis and recovery, microparticles were characterized by dynamic light scattering (DLS), scanning and transmission electron-microscopy (SEM/TEM), quantitative-analysis, Fourier-transform infrared-spectroscopy (FTIR), X-ray diffraction studies (XRD), differential scanning-calorimetry (DSC), thermogravimetric analysis (TGA), in vitro cytotoxicity assay, antibacterial-activity, pH analysis and drug-release kinetics. (**c**) Representative SEM image of the synthesized spherical-shaped uniformly distributed CHX-loaded/Ca(OH)_2_ microparticles.

The particulars of the synthesis procedure of the CHX-loaded/Ca(OH)_2_ and the unloaded [Ca(OH)_2_/Blank] microparticles are pictorially represented in Figure 1a. The Ca(OH)_2_/Blank and the CHX-loaded/Ca(OH)_2_ microparticles were prepared by a modified aqueous chemical-precipitation method [21]. Briefly, the aqueous solutions containing 0.3 mol/L of CaCl_2_·2H_2_O and 0.6 mol/L of NaOH were prepared separately, with the supplementation of 0.15% of Triton X-100 to these initial solutions. Different amounts of 25 and 50 mg of chlorhexidine (CHX) was added to the formulated CaCl_2_·2H_2_O solution and mixed well. The modified CaCl_2_ solution was added drop-wise into the aqueous NaOH at ~50 °C, forming CHX-loaded/Ca(OH)_2_ microparticles at formulations of CHX:Ca(OH)_2_/25 mg and CHX:Ca(OH)_2_/50 mg respectively. A white chalky precipitate was obtained after 1 h. The precipitate was recovered by centrifugation, vacuum desiccated to minimize the extent of carbonation process [22] and stored at 4 °C for further investigation and characterization. The blank-microparticles were synthesized in the same aforesaid method, without the supplementation of CHX.

### 2.3. Morphological Features

The fabricated microparticles were characterized, in vitro, by various techniques indicated in Figure 1b,c. The Ca(OH)_2_/Blank and the CHX-loaded/Ca(OH)_2_ microparticles (at formulations of CHX:Ca(OH)_2_/25 mg and CHX:Ca(OH)_2_/50 mg) were characterized by dynamic light scattering (DLS) technique (Malvern Mastersizer Nano ZS, UK) for estimation of the *z*-average diameter, surface-charge and poly-dispersity index (PDI). Microparticles were diluted in distilled water [in the ratio of 1/50 (*wt/v*)] and measurements were made at 37 °C (n = 9). The particle shape, structure, morphology, surface texture, occurrence of the aggregation phenomena, the elemental composition and distribution on the surface of the synthesized Ca(OH)_2_/Blank and the CHX-loaded/Ca(OH)_2_ microparticles were determined by SEM (Joel FESEM JSM-6700F, Japan) equipped with energy-dispersive X-ray spectroscopy (EDX-SEM; an Oxford/INCA EDS) (n = 5). Prior to examination, dried particles were spread on double-coated carbon adhesive tapes fixed on metal stubs and gold sputter-coated under an argon atmosphere (BAL-TEC, SCD 005 Sputter Coater, Scotia, NY, USA) for SEM imaging at an accelerating voltage of 10 kV. TEM examination of the Ca(OH)_2_/Blank and the CHX-loaded/Ca(OH)_2_ microparticles was performed using the Joel-JSM-1010 (Japan). A drop of microparticles diluted in distilled water [in the ratio of 1:50 (*wt/v*)] were placed on carbon-coated copper grids and dried overnight. The dried samples were then examined using TEM.

### 2.4. Encapsulation-Efficiency and Drug-Loading

The percentage (%) drug encapsulation-efficiency (EE) of the CHX-loaded/Ca(OH)_2_ microparticles (at formulations of CHX:Ca(OH)_2_/25 mg and CHX:Ca(OH)_2_/50 mg) was determined by indirect-assay method. Microparticles were washed with distilled water, centrifuged and the supernatant was collected. Based on the standard curve plotted earlier, the amount of non-encapsulated (free) CHX in the supernatant was measured spectrophotometrically at 289 nm (UV-1700 Pharma Spec UV-VIS Spectrophotometer, Shimadzu, Japan) (n = 7). From the above estimated value, the amount of encapsulated CHX was determined. The percentage (%) of drug encapsulation-efficiency (EE) and drug-loading (DL) were calculated using Equations (1) and (2).

(1) $$\mathrm{Encapsulation}\;\mathrm{Efficiency}\;\left(\%\right)=\frac{\mathrm{Mass}\;\mathrm{of}\;\mathrm{drug}\;\mathrm{in}\;\mathrm{microparticles}\;\times \;100}{\mathrm{Mass}\;\mathrm{of}\;\mathrm{drug}\;\mathrm{added}}$$

(2) $$\mathrm{Drug}\;\mathrm{loading}\;\left(\%\right)=\frac{\mathrm{Mass}\;\mathrm{of}\;\mathrm{drug}\;\mathrm{in}\;\mathrm{microparticles}\;\times \;100}{\mathrm{Mass}\;\mathrm{of}\;\mathrm{particles}\;\mathrm{recovered}}$$

### 2.5. Spectral and Thermal Analyses

The IR spectra of the Ca(OH)_2_/Blank and the CHX-loaded/Ca(OH)_2_ microparticles (at formulations of CHX:Ca(OH)_2_/25 mg and CHX:Ca(OH)_2_/50 mg) were recorded using Perkin Elmer Spectrum RX-I (Waltham, MA) to detect the structural-features and confirm inclusion of CHX in the CHX-loaded/Ca(OH)_2_ microparticles. Powdered microparticles samples were mixed with KBr and pressed to pellets for measurements. Samples were run in duplicates and the spectra were obtained at frequency range of 4000 to 400 cm^−1^ at 4 cm^−1^ resolution. The X-ray powder diffraction (XRD) measurements were performed to determine the crystalline phases of the synthesized Ca(OH)_2_/Blank and the CHX-loaded/Ca(OH)_2_ microparticles (at the formulations of CHX:Ca(OH)_2_/25 mg and CHX:Ca(OH)_2_/50 mg) and confirm the presence of CHX in all the CHX:Ca(OH)_2_ formulations. The data were collected for the powdered samples under laboratory conditions (T = 20 °C, relative humidity RH = 40%), using a Rigaku Miniflex X-ray diffractometer (Tokyo, Japan) using Cu-Kα radiation (kα1 = 1.542 Å). Data were collected over the 2θ range with a step size of 0.02°. The thermal stability of the Ca(OH)_2_/Blank and the CHX-loaded/Ca(OH)_2_ microparticles (at formulations of CHX:Ca(OH)_2_/25 mg and CHX:Ca(OH)_2_/50 mg) was verified by thermogravimetric analysis (TGA), using a TA Instrument Discovery TGA Thermogravimetric analyzer (New Castle, DE, USA). Approximately 10 mg of the samples were placed in alumina crucibles and heated from room temperature to 800 °C at a heating rate of 10 °C min^−1^ under nitrogen atmosphere. Differential scanning calorimetry (DSC) thermograms of the Ca(OH)_2_/Blank and the CHX-loaded/Ca(OH)_2_ microparticles were obtained using a Mettler Toledo (DSC-1 system, Greifensee, Switzerland) for determining the physical state of the drug present in the microparticles formulations. About 2 mg of the powdered samples placed in an aluminium pan were heated from room temperature to 400 °C under nitrogen atmosphere at a heating rate of 10 °C min^−1^.

### 2.6. Evaluation of pH

For determining the pH, about 15 mg of the microparticles and commercial Ca(OH)_2_ powder was suspended in 10 mL of distilled water and maintained at 37 °C. A standard Dycal disc was prepared according to the manufacturer’s instructions, allowed to set and ground to fine powder using mortar and pestle. Further, Dycal samples were maintained under same conditions as that of the microparticles. The pH values were recorded using Thermo Scientific^TM^ Orion^TM^ Star A211 pH Benchtop Meter (CO, Fort Collins, USA) for 0 h, 24 h, 7 days and 15 days (n = 13).

### 2.7. Cytotoxicity Assay

The in vitro cytotoxicity assay of the Ca(OH)_2_/Blank and the CHX-loaded/Ca(OH)_2_ microparticles (at formulations of CHX:Ca(OH)_2_/25 mg and CHX:Ca(OH)_2_/50 mg) was assessed by Vybrant MTT Cell Proliferation Assay Kit purchased from Thermo Fisher Scientific (Waltham, MA, USA). Human mesenchymal stem cells (hMSCs) (Cat # PT-2501) were seeded in 96-well plates at 1 × 10^4^ cells per well. Hundred microliters of suspended cells were added in Mesenchymal Stem Cell Growth Medium (MSCGM^TM^)/well (Lonza, Basel, Switzerland). After 24 h of plating, the cells were exposed to different concentrations (20, 60 and 100 μg/mL) of Dycal, pure CHX, commercial Ca(OH)_2_ powder, Ca(OH)_2_/Blank and CHX-loaded/Ca(OH)_2_ microparticles (at formulations of CHX:Ca(OH)_2_/25 mg and CHX:Ca(OH)_2_/50 mg). Dycal was prepared according to manufacturer’s instructions, allowed to set and powdered (as mentioned previously). Dried powders of pure CHX, Dycal and microparticles were suspended in the culture medium (i.e. at concentration of 20 μg/mL, 20 μg of nanoparticles were suspended in 1 mL of the culture medium). About 30 μL of this suspension was directly added to the cultured cells. The cells were cultured for 24 h with the microparticles. Untreated-control cultures (cells and culture medium only) were also maintained in the same conditions. The cells were washed with PBS and subjected to MTT assay. Briefly, the cells were treated with 12 mM of 3-(4,5-dimethylthiazol-2-yl)-2,5-diphenyltetrazolium bromide (MTT) stock solution in each well. After a 4 h incubation at 37 °C, 100 μL of SDS-HCl solution was added to each well and mixed thoroughly with the pipette. The microplate was incubated at 37 °C in a humidified chamber for 3 h. Each sample was mixed again using a pipette and the absorbance was read at λ = 570 nm in a microplate reader. The experiments were performed in triplicates (n = 7) and the percentage (%) cell viability was determined from the absorbance by taking the values of untreated group as 100%.

### 2.8. Agar-Diffusion Bacterial Inhibition Zone Test

*Streptococcus mutans* ATCC UA159 and *Enterococcus faecalis* ATCC 29212 were the strains used in this study. Bacteria from frozen stock was recovered in 5 mL of brain–heart infusion (BHI) broth and cultured aerobically at 37 °C. From the overnight culture, an inoculum of 0.5 at OD600 (equivalent to 10^8^ bacteria/mL) was prepared in fresh BHI. About 100 μL of each bacterial suspension was freshly seeded on BHI agar plate, swabbed and dried for 20 min. In this method, the agar disk and well-diffusion procedures have been combined. Holes were cut in the agar plates and 30 μL of Ca(OH)_2_/Blank, CHX:Ca(OH)_2_ microparticles (at formulations of CHX:Ca(OH)_2_/25 mg and CHX:Ca(OH)_2_/50 mg) and commercial Ca(OH)_2_ powder were placed in the holes. About 20 μL of the corresponding positive-control CHX concentrations (dissolved in acetone) were deposited on the filter paper-disks, air-dried and evenly spaced on the prepared bacterial agar petri-dishes. Dycal was placed inside the well immediately after mixing both the catalyst and base pastes, in order for the paste to set inside the well. All plates were incubated at 37 °C for 48 h (n = 13). The diameters of the inhibition-zones were measured in centimeters (cm) with a slide gauge.

### 2.9. Preparation of Dentin-Specimens for Microparticles Application

**Figure 2:**
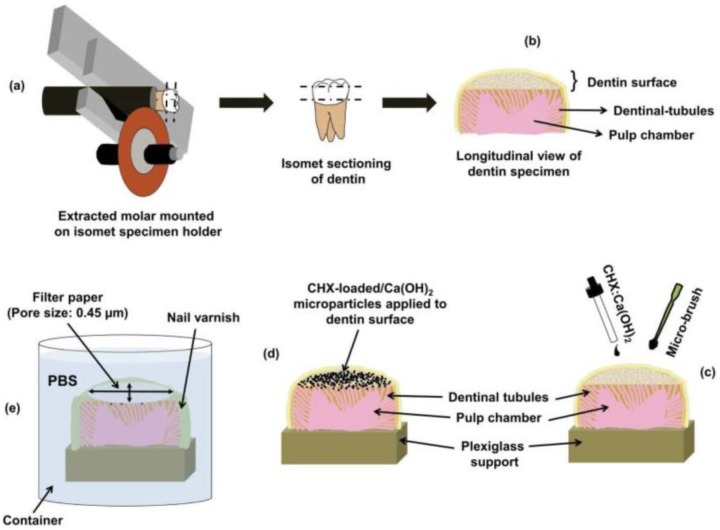
(**a**) Schematic sketch demonstrating the isomet sectioning procedure of extracted human molars for obtaining dentin-specimens used for microparticles delivery, the ex vivo drug-release experiments, and the confocal imaging for Live/Dead assay. The occlusal enamel surface was removed using low-speed diamond saw exposing the superficial-dentin approximately 3 mm beneath the DEJ (deep dentin). The dentin specimens were prepared by cutting parallel to the exposed dentin surface, about 0.5 mm above the CEJ, under running water. (**b**) After sectioning, the dentin specimens were wet-ground using 600 through 4000 grit-size silicon-carbide papers. The prepared dentin-surfaces were cleaned ultrasonically for 10 min and rinsed with distilled water. (**c**) Representative sketch showing the procedure of treatment of the prepared dentin-surface with 30 μL of microparticles suspended in distilled water (at the working ratio of 1/1 (*wt/v*)), applied drop-by-drop for 60 s followed by gentle spreading on the surface by micro-brush for 5 s. Following application, the dentin-surface was gently air-blown for 3 s and the excess water was blot-dried by absorbent paper. (**d**) The CHX-loaded/Ca(OH)_2_ microparticles attached to the exposed dentin-surface following treatment. (**e**) For characterization of CHX-release profiles from microparticles delivered to the dentin-substrates, oval-shaped filter-paper strips (Whatman PTFE membrane filters; average pore size: 0.45 μm) (8 mm × 5 mm) were placed on the exposed surfaces of the microparticles-loaded dentin-specimens, just enough to cover the surface. The other exposed areas that are not covered by the filter paper were sealed with a water-proof nail varnish to standardize the area for CHX-release. The entire set-up was placed inside a container filled with 10 mL PBS for determining the ex vivo release of CHX into the surrounding release media over pre-determined time-intervals. The amount of CHX liberated from dentin into the surrounding PBS release medium was analyzed spectrophotometrically. N.B: The longitudinal section of the prepared dentin specimen displayed in the schematic sketch is only used for demonstration purpose. It is important to note that no longitudinal sectioning was performed and the dentin specimens were used as an intact specimen throughout the ex vivo experiments. * DEJ: Dentino-enamel junction; CEJ: Cemento-enamel junction.

Non-carious and non-restored human molars (primarily extracted for clinical reasons) from patients of age range 21–35 years were chosen as ex vivo models for investigating the application of the CHX-loaded/Ca(OH)_2_ to dentin-substrates. Generic ethical approval and consent has been acquired for research to be conducted on the collected teeth from the Institutional Review Board of the National University of Singapore. The collected teeth were stored in 0.5% Chloramine-T solution for 2 weeks after which they were stored in distilled water at 4 °C and used within two months from the extraction time. A low-speed diamond saw (Buehler, Lake Bluff, IL, USA) was used to obtain flat, transversely cut dentin-specimens under water-coolant as shown in Figure 2a,b. The specimens were then wet-ground under water-coolant with 600 through 4000 grit-size silicon-carbide abrasive papers (Carbimet; Buehler Ltd., Lake Bluff, IL, USA) using a micro-grinder/polisher machine (Phoenix Beta Polisher/Grinder, Phoenix, AZ, USA). The dentin-specimens were ultrasonically cleaned for 10 min and rinsed with distilled water. Remnants of the pulp-tissues were removed by gentle excavation followed by normal saline irrigation.

### 2.10. Microparticles Application to Surface-Prepared Dentin-Substrates

The prepared dentin-specimens were randomly grouped to be treated with: (i) Ca(OH)_2_/Blank (control), (ii) CHX:Ca(OH)_2_/25 mg microparticles and (iii) CHX:Ca(OH)_2_/50 mg microparticles. Water was used as the microparticles’ carrier at the microparticles/carrier working ratio of 1/1 (*wt/v*). An amount of 30 μL of microparticles/carrier suspension was applied drop-by-drop to the prepared dentin-specimens for 60 s followed by gentle spreading on the surface by micro-brush rubbing for 5 s as shown in Figure 2c,d. After microparticles application, the dentin-substrates were gently air-blown for 3 s and the excess water was blot-dried by absorbent paper. The dentin-specimens treated with the microparticles were prepared for SEM examination.

### 2.11. Biofilm Formation and Live/Dead Cell Assay

For biofilm formation, the inoculation of *Streptococcus mutans* (ATCC UA159) and *Enterococcus faecalis* (ATCC 29212) suspensions adjusted to OD 0.5 at 600 nm (equivalent to 10^8^ bacteria/mL) were prepared as mentioned previously. Dentin-specimens treated with the microparticles were used as substrates for biofilm attachment and growth. Dentin-specimens were placed in 24-well plate (one-specimen per well) inoculated with 1 mL of bacterial suspension cells at 37 °C in 5% CO_2_ and left undisturbed for 3 days (n = 5). Every 24 h the samples were replenished with BHI medium to remove non-adherent cells.

The *S. mutans* and *E. faecalis* biofilms attached on the microparticle-treated dentin specimens were stained with Film Tracer^TM^ LIVE/DEAD Biofilm Viability kit (Life Technologies-Invitrogen, Grand Island, NY, USA) using SYTO-9 dye and propidium iodide (PI) (ratio of 1:1), according to the manufacturer’s instructions. SYTO-9 stains metabolically active cells that emits a green fluorescence (excitation/emission wavelength of 480/500 nm) while PI stains dead cells giving a red fluorescence (excitation/emission wavelength of 490/635 nm) [23]. Stained dentin-specimens were rinsed with 1 mL of sterile distilled water. Samples were later imaged using a 60× water immersion lens fitted to a confocal laser scanning microscope (Olympus Fluoview 1000; Olympus, Tokyo, Japan). The fluorescence signal intensities of biofilm were analyzed with IMARIS 6.3 software (Biplanes Scientific, Zurich, Switzerland).

### 2.12. CHX Release-Profiles

The in vitro release-profiles of CHX were determined by suspending 5 mg of dried CHX-loaded/Ca(OH)_2_ microparticles (at formulations of CHX:Ca(OH)_2_/25 mg and CHX:Ca(OH)_2_/50 mg) in 10 mL of PBS at pH 7.4 in a magnetic stirrer (Thermo Scientific™ Cimarec™ Digital Hotplates, Stockton, CA, USA) maintained at 37 °C. The amount of CHX released from the microparticles were analyzed spectrophotometrically (UV-1700 Pharma Spec UV-VIS Spectrophotometer, Shimadzu, Japan) at 289 nm at predetermined time-intervals up to 15 days (n = 13). For the ex vivo characterization of CHX-release profiles from CHX:Ca(OH)_2_/25 mg and CHX:Ca(OH)_2_/50 mg microparticles delivered to dentin-substrates; oval-shaped filter-paper strips (Whatman PTFE membrane filters; pore size:0.45 μm) (8 mm × 5 mm) were prepared and placed on the exposed surfaces of the microparticles-loaded dentin-substrates, just enough to cover the surface. The other exposed areas not covered by the filter paper were sealed with water-proof nail varnish in order to standardize the area for CHX-release. The entire set-up was placed inside a container having 10 mL of PBS (pH 7.4) (Figure 2e). The amount of CHX released from microparticles applied to the dentin-substrates through the filter paper to the external PBS release medium was detected by analyzing the PBS spectrophotometrically over pre-determined time-intervals (n = 13). Experiments were performed in duplicates and the % CHX release was calculated as mean (±standard deviation).

### 2.13. Statistical Analysis

Data were presented as mean ± standard deviation values and the statistical analysis was done using ANOVA followed by Tukey–Kramer post-hoc test at a chosen significance-level of *p* ≤ 0.05.

## 3. Results

### 3.1. Morphological Features

The *z*-average diameter, surface-charge and PDI of the Ca(OH)_2_/Blank and CHX-loaded/Ca(OH)_2_ microparticles determined by DLS are indicated in Table 1. The synthesis of the Ca(OH)_2_/Blank and the CHX-loaded/Ca(OH)_2_ microparticles by aqueous chemical-precipitation method has resulted in particle-size ranging from 1.4 ± 0.3 μm to 5.3 ± 0.2 μm. A significant decrease in particle-size was observed upon incorporation of different amounts of CHX (25 and 50 mg). Interestingly, the low ζ-potential of Ca(OH)_2_/Blank (+2.19 ± 0.4 mV) was raised to +23.52 ± 4.5 mV and +35.97 ± 8.6 mV respectively, upon increasing CHX incorporation. The microparticles exhibited PDI values ranging from 0.789 ± 0.038 to 0.319 ± 0.093, exhibiting a negative-shift with escalating addition of CHX and positive-shift with the increasing particle size.

**Figure 3:**
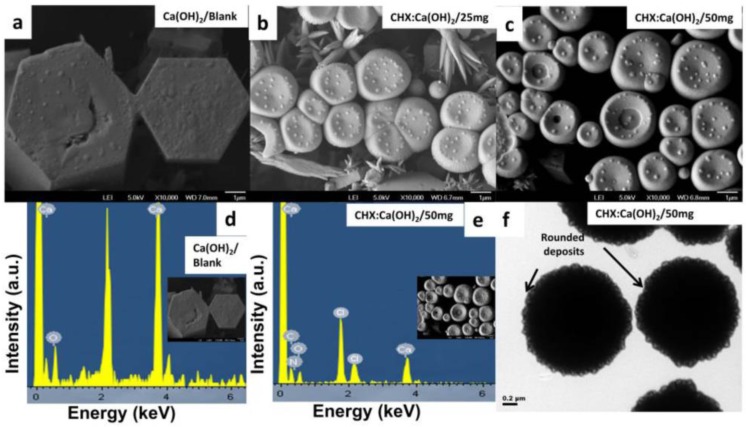
Representative SEM images showing the morphology of (**a**) the unloaded Ca(OH)_2_ microparticles [Ca(OH)_2_/Blank]; (**b**,**c**) the CHX-loaded/Ca(OH)_2_ microparticles at the formulations of CHX:Ca(OH)_2_/25 mg and CHX:Ca(OH)_2_/50 mg respectively. Ca(OH)_2_/Blank-microparticles (**a**) were hexagonal in shape; whereas the CHX:Ca(OH)_2_/25 mg (**b**) and the CHX:Ca(OH)_2_/50 mg (**c**) microparticles exhibited spherical shape with distinctly visible “rounded deposits” on the microparticles surface (**d**,**e**). Representative EDX-SEM elemental-composition spectral characterization of the (**d**) the unloaded Ca(OH)_2_ microparticles [Ca(OH)_2_/Blank] showing only the Ca/O signals confirmed the presence of Ca(OH)_2_ in their structure and (**e**) the CHX:Ca(OH)_2_/50 mg displaying the presence of the elements chlorine, nitrogen and carbon, confirmed the presence of these elements constituting CHX, apart from the Ca/O signals which again substantiated the presence of Ca(OH)_2_. (**f**) Selected high magnification TEM image of the CHX:Ca(OH)_2_/50 mg microparticles confirming the spherical morphology and the prominent “rounded-deposits” seen evenly distributed on the surface of microparticles.

Representative SEM and TEM micrographs of the unloaded Ca(OH)_2_ [Ca(OH)_2_/Blank] and the CHX-loaded/Ca(OH)_2_ microparticles are shown in Figure 3a–c,f indicating successful synthesis of microparticles with less agglomeration. The Ca(OH)_2_/Blank-microparticles displayed uniform particle-distribution with hexagonally-plated regularly-shaped appearance (Figure 3a). Upon addition of CHX, the CHX-loaded/Ca(OH)_2_ microparticles were spherical-shaped with smooth texture with their size smaller than that of the unloaded Ca(OH)_2_/Blank microparticles (Figure 3b,c and Table 1). An even dent-like impression was observed on the surface of CHX-loaded/Ca(OH)_2_ microparticles as shown in Figure 3b,c. Representative EDX-SEM elemental-composition spectral characterization of the Ca(OH)_2_/Blank showed only the Ca/O signals confirming the presence of Ca(OH)_2_ (Figure 3d) whereas that of the CHX:Ca(OH)_2_/50 mg-microparticles consisted of chlorine, nitrogen and carbon (the elements confirming the presence of CHX) apart from the Ca/O signals (Figure 3e). Therefore, EDX data confirmed the presence of CHX on the surface of the CHX-loaded/Ca(OH)_2_ formulations. Similar information on the morphology of the Ca(OH)_2_ particles have been obtained from TEM investigations, revealing numerous “rounded-deposits” on the microparticle surface (Figure 3f). TEM results were in agreement with the SEM and DLS data.

### 3.2. Encapsulation-Efficiency and Drug-Loading

The percentage (%) of drug encapsulation-efficiency (EE), drug-loading (DL) and particle-recovery of the Ca(OH)_2_/Blank and the CHX-loaded/Ca(OH)_2_ microparticles (at formulations of CHX:Ca(OH)_2_/25 mg and CHX:Ca(OH)_2_/50 mg) are shown in Table 1. The % EE has shown a significant increase from 39.16% ± 1.6% to 62.34% ± 2.4% with the increasing supplementation of CHX from 25 to 50 mg. Initial CHX addition of 25 mg resulted in a % DL of 8.80% ± 6.1% whereas further increase in CHX-incorporation has increased the % DL reaching a value of 20.53% ± 3.4%. A positive-shift in the % EE values could be noticed with a subsequent increase in the % DL. Therefore, the highest %EE (62.34% ± 2.4%) was achieved with a DL of 20.53% ± 3.4% (Table 1). The percentage (%) microparticle recovery values of the Ca(OH)_2_/Blank has shown to be 40.26% ± 2.6% whereas CHX-inclusion resulted in values of about 38.05% ± 3.5% and 30.78% ± 1.9% respectively.

### 3.3. Spectral Analyses

**Figure 4:**
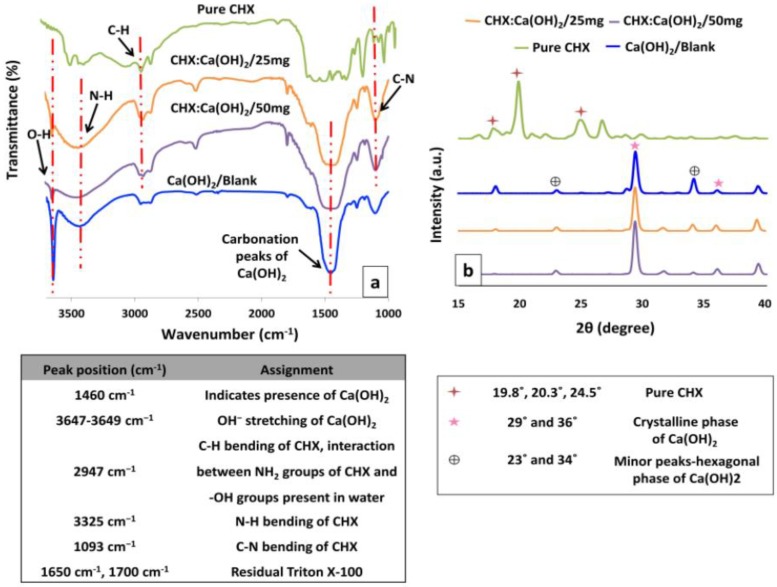
(**a**) Representative FTIR showing the vibrational-bands obtained for the pure Chlorhexidine (CHX), the Ca(OH)_2_/Blank microparticles and the CHX-loaded/Ca(OH)_2_ microparticles at the formulations of CHX:Ca(OH)_2_/25 mg and CHX:Ca(OH)_2_/50 mg respectively. The FTIR data confirms the evidence of CHX inclusion in the CHX-loaded/Ca(OH)_2_ microparticles. The vibrational-bands observed at 1460 cm^−1^ confirms the presence of Ca(OH)_2_ and the presence of peak at 3647–3649 cm^−1^ attribute to OH^−^ stretching of the solid Ca(OH)_2_ crystals in all formulations of CHX-loaded/Ca(OH)_2_ microparticles. The fingerprint peaks specific to CHX shows characteristic peaks at 2947 cm^−1^, 3325 cm^−1^ and 1093 cm^−1^ for C–H, N–H and C–N respectively, observed in both formulations of CHX-loaded/Ca(OH)_2_. The interaction between the amine (–NH_2_) groups in CHX and the hydroxyl (–OH) groups present in Ca(OH)_2_ has resulted in the binding of CHX to the Ca(OH)_2_ carrier. Accordingly, the N-H bend (3325 cm^−1^) was notably flatter/wider and C–N peak (1093 cm^−1^) became deeper upon CHX inclusion. The positions of the FTIR peak frequencies and their corresponding assignment in the Ca(OH)_2_/Blank, pure Chlorhexidine (CHX) and CHX-loaded/Ca(OH)_2_ microparticles at formulations of CHX:Ca(OH)_2_/25 mg and CHX:Ca(OH)_2_/50 mg respectively have been indicated in the table *(left)*. (**b**) Representative X-ray diffractograms (XRD) of pure Chlorhexidine (CHX), the Ca(OH)_2_/Blank microparticles, and the CHX-loaded/Ca(OH)_2_ microparticles at the formulations of CHX:Ca(OH)_2_/25 mg and CHX:Ca(OH)_2_/50 mg are displayed respectively. The typical XRD peaks of Ca(OH)_2_ present the diffraction angles at 29° and 36° indicating the crystalline phase of Ca(OH)_2_ in the all formulations. Pure CHX showed clear peaks at 2θ of 19.8°, 20.3° and 24.5° and the absence of these characteristic peaks in the CHX-loaded/Ca(OH)_2_ formulations confirmed uniform CHX dispersion in the structure of the CHX-loaded/Ca(OH)_2_ microparticles. The positions of the XRD peaks and their corresponding assignment in the Ca(OH)_2_/Blank microparticles, pure Chlorhexidine (CHX), CHX-loaded/Ca(OH)_2_ microparticles have been indicated in the table *(right)*.

The FTIR spectra of the Ca(OH)_2_/Blank and the CHX-loaded/Ca(OH)_2_ microparticles are shown in Figure 4a. The vibrational-bands observed at 1460 cm^−1^ confirms the presence of Ca(OH)_2_ in all CHX:Ca(OH)_2_ formulations and corresponds to slight carbonation of Ca(OH)_2_ due to mixing with KBr powder in the air as mentioned in previous studies [24]. The sharp peak at 3647–3649 cm^−1^ attribute to OH^−^ stretching of the solid Ca(OH)_2_ crystals and the intensity of this peak has shown to diminish upon escalating CHX-incorporation to the CHX:Ca(OH)_2_ microparticles. The interaction between the amine (-NH_2_) groups in CHX and the hydroxyl (-OH) groups present in Ca(OH)_2_ has resulted in the binding of CHX to the Ca(OH)_2_ carrier [25]. The FTIR spectra of pure CHX has been shown for reference exhibiting characteristic peaks at 2947 cm^−1^, 3325 cm^−1^ and 1093 cm^−1^ for C-H, N-H and C-N respectively. The above-mentioned fingerprint peaks specific to CHX can be observed in all the CHX-loaded/Ca(OH)_2_ formulations as shown in Figure 4a. These results confirm effective inclusion of CHX in the Ca(OH)_2_ carrier. Since this procedure involves preparation of CHX-loaded/Ca(OH)_2_ microparticles by a wet method, few other peaks at 1650 cm^−1^, 1700 cm^−1^ have also been detected, that could be due to the presence of residual Triton X-100 in the reaction system [26]. The characteristic peak of both CHX and Ca(OH)_2_ seen in the FTIR spectra of the CHX:Ca(OH)_2_ formulations indicates the presence of CHX in CHX:Ca(OH)_2_ particles and re-confirms the results obtained by SEM and TEM.

The XRD profiles of the Ca(OH)_2_/Blank and the CHX:Ca(OH)_2_ microparticles are shown in Figure 4b. The typical XRD peaks of Ca(OH)_2_ present the diffraction angles at 29° and 36° indicating the crystalline phase of Ca(OH)_2_ in all the formulations [21]. However, some minor peaks representing the hexagonal phases at 23° and 34° were also noticed [27]. Pure CHX has shown clear peaks at 2θ of 19.8°, 20.3° and 24.5° (shown for reference). The absence of these characteristic peaks specific to CHX at 19.8°, 20.3° and 24.5° in the CHX:Ca(OH)_2_ formulations confirmed significant incorporation and effective loading of CHX in the structure of the formed CHX:Ca(OH)_2_ microparticles. The uniform dispersion of CHX in CHX:Ca(OH)_2_ carrier has contributed to the disappearance of these peaks. It can also be observed that except for Ca(OH)_2_ and CHX no obvious peak has resulted indicating reproducibility and uniqueness of the synthesis procedure. XRD results confirmed the successful inclusion of CHX in the CHX:Ca(OH)_2_ formulations and are in agreement with the data obtained from FTIR analysis.

### 3.4. Thermal Properties

**Figure 5:**
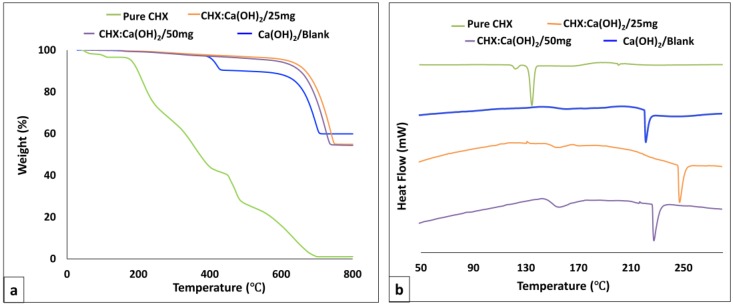
(**a**) Representative Thermogravimetric Analysis (TGA) profiles displaying the spectra of the pure Chlorhexidine (CHX), the unloaded Ca(OH)_2_ microparticles [Ca(OH)_2_/Blank], and the CHX-loaded/Ca(OH)_2_ microparticles at the formulations of CHX:Ca(OH)_2_/25 mg and CHX:Ca(OH)_2_/50 mg respectively. The Ca(OH)_2_/Blank microparticles showed decomposition at approximately 350–400 °C. Pure CHX has demonstrated weight-loss at ~100 °C whereas its entrapment in Ca(OH)_2_ microparticles shifted degradation to higher temperatures (~450–500 °C). Therefore, entrapment procedure has resulted in structural changes in CHX-loaded/Ca(OH)_2_ microparticles (**b**) Representative DSC thermograms showing the spectra of the pure Chlorhexidine (CHX), the Ca(OH)_2_/Blank microparticles, and the CHX-loaded/Ca(OH)_2_ microparticles at the formulations of CHX:Ca(OH)_2_/25 mg and CHX:Ca(OH)_2_/50 mg respectively. The endotherm peaks of Ca(OH)_2_/Blank were observed at ~222.38 °C. Upon CHX incorporation, CHX endotherm peaks were displaced to a temperature of ~250.28 °C and ~235.40 °C in CHX:Ca(OH)_2_/25 mg and CHX:Ca(OH)_2_/50 mg, respectively, confirming the presence of CHX in these formulations. Absence of the detectable CHX domains (~136 °C) in the spectra of microparticles indicated uniform CHX dispersion in the Ca(OH)_2_ carrier.

Representative TGA thermograms showing the percentage weight loss of the Ca(OH)_2_/Blank, the CHX-loaded/Ca(OH)_2_ microparticles and pure CHX are shown in Figure 5a. It has been widely recognized that the decomposition temperature of Ca(OH)_2_ occurs between 350 °C and 550 °C [28]. The TGA curve of the pure CHX (shown for reference), demonstrated mass-loss around 100 °C as indicated in previous studies [29]. From the obtained TGA curves, the Ca(OH)_2_/Blank microparticles has undergone decomposition at approximately 350–400 °C whereas CHX encapsulation shifted the decomposition to higher temperatures (~450–500 °C). It could also be inferred that the entrapment of CHX in Ca(OH)_2_ microparticles resulted in structural changes in the microparticles. DSC studies were analyzed to understand the nature of the drug in the particle carrier (Figure 5b). The physical state of CHX in the CHX-loaded/Ca(OH)_2_ microparticles would also influence its release characteristics. To probe this effect, DSC measurements were performed on the Ca(OH)_2_/Blank, the CHX-loaded/Ca(OH)_2_ microparticles (at formulations of CHX:Ca(OH)_2_/25 mg and CHX:Ca(OH)_2_/50 mg) and pure CHX. From the DSC curves displayed in Figure 5b, the endotherm peaks of Ca(OH)2/Blank were observed at ~222.38 °C. Upon incorporation of CHX, the endothermic peaks of CHX:Ca(OH)_2_/25 mg and CHX:Ca(OH)_2_/50 mg have been displaced to a temperature of ~252.28 °C and ~228.40 °C respectively. The endotherm of pure CHX (~135.45 °C) was not detected in the microparticle formulations. Absence of the detectable CHX domains in the spectra of CHX:Ca(OH)_2_ formulations indicated uniform CHX dispersion in the CHX:Ca(OH)_2_ carrier and therefore, corroborated the XRD findings. Hence, the displacement of the endothermic peaks of CHX-loaded/Ca(OH)_2_ microparticles mainly indicates successful CHX-incorporation in the formulations.

### 3.5. Evaluation of pH

**Figure 6:**
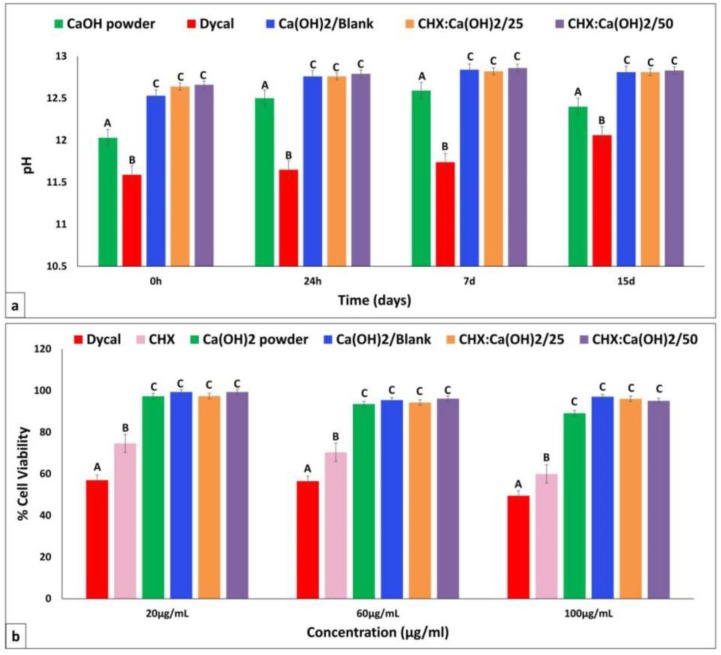
(**a**) Mean ± standard deviation of the measured pH values of the prepared Ca(OH)_2_/Blank microparticles, CHX-loaded/Ca(OH)_2_ microparticles (at formulations of CHX:Ca(OH)_2_/25 mg and CHX:Ca(OH)_2_/50 mg), Dycal and the commercial Ca(OH)_2_ powder determined at 0 h, 24 h, 7 days and 15 days respectively. The pH values of the prepared microparticles were consistently high ranging from ~12.5 to ~13.06 at all specified time points; whereas that of the commercial Ca(OH)_2_ powder was comparatively lower ranging from ~12 to 12.5. No significant difference was observed between pH of the Ca(OH)_2_/Blank and the CHX-loaded/Ca(OH)_2_ microparticles. Dycal demonstrated lowest pH with values ranging from ~11.59 to ~12.06. (**b**) Mean ± standard deviation of the percentage (%) cell viability of human mesenchymal stem cells (hMSCs) showing low cytotoxicity profile (>90%) upon 24 h of exposure to the different concentrations (20, 60 and 100 μg/mL) of the Ca(OH)_2_/Blank microparticles, the CHX-loaded/Ca(OH)_2_ microparticles, and the commercial Ca(OH)_2_ powder. On the contrary, exposure to Dycal and pure CHX has reduced the viability of cells. * Groups with different alphabets are statistically significant (*p* ≤ 0.05) within each time point and concentration for pH and cell viability respectively. ** Statistical analysis was done with one-way ANOVA followed by Tukey–Kramer post-hoc test.

The pH values of Ca(OH)_2_/Blank and CHX-loaded/Ca(OH)_2_ microparticles (at formulations of CHX:Ca(OH)_2_/25 mg and CHX:Ca(OH)_2_/50 mg) remained consistently high, ranging from ~12.5 to 13.06 up to 15 days as displayed in Figure 6a. There was no significant difference between pH of Ca(OH)_2_/Blank and CHX-loaded/Ca(OH)_2_ microparticles at all determined time points. The pH of commercial Ca(OH)_2_ powder was slightly lower than the microparticles (ranging from ~12 to 12.5) but higher than that of Dycal, which exhibited lowest pH values ranging from ~11.59 to 12.06.

### 3.6. Cytotoxicity Assay

The cell-viability of the Ca(OH)_2_/Blank and CHX-loaded/Ca(OH)_2_ microparticles (at formulations of CHX:Ca(OH)_2_/25 mg and CHX:Ca(OH)_2_/50 mg) was >90% at all tested concentrations of 20, 60 and 100 μg/mL as shown in Figure 6b. Therefore, particles showed low toxicity to hMSCs at the proposed concentrations. Hence Ca(OH)_2_/Blank and both formulations of CHX-loaded/Ca(OH)_2_ microparticles are relatively biocompatible to hMSCs during the 24 h of exposure. The commercial Ca(OH)_2_ powder also promoted cell proliferation resulting in high cell-viability values (>90%). However, pure CHX has reduced the viability of the cells in comparison to the microparticles. Dycal has shown strong cytotoxicity at all concentrations resulting in in <60% viable cells.

### 3.7. Bacterial Inhibition Zone

The diameters of zones of inhibition for *E. faecalis* and *S. mutans* in the presence of Ca(OH)_2_/Blank and CHX-loaded/Ca(OH)_2_ microparticles (at formulations of CHX:Ca(OH)_2_/25 mg and CHX:Ca(OH)_2_/50 mg) are shown in Table 2. CHX-loaded/Ca(OH)_2_ microparticles have shown activity against both the strains (after 48 h) although pure unencapsulated CHX has shown to be more effective than the synthesized CHX-loaded/Ca(OH)_2_ microparticles. Dycal and the commercial Ca(OH)_2_ powder have exhibited reduced antibacterial efficacy than the pure CHX; and the Ca(OH)_2_/Blank and CHX-loaded/Ca(OH)_2_ microparticles.

### 3.8. Microparticles Application to Dentin-Substrates

**Figure 7:**
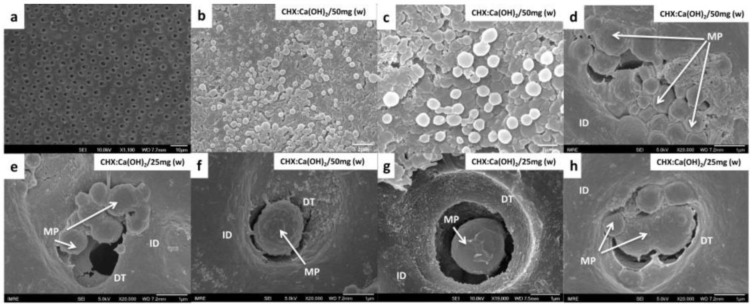
(**a**) Representative SEM image of the ultra-high polished specimens showing the dentinal tubules structure of the deep dentin (scale: 10 µm). (**b**,**c**) Selected SEM images, at different magnifications, showing the formation of an intact layer of evenly-distributed spherical microparticles attached and covering the prepared dentin surface (scale: 2 µm and 1 µm respectively). This indicates successful attachment of microparticles on the exposed dentin-surfaces. (**d**–**g**) Selected high magnification SEM images, at different magnifications, showing the ability of the microparticles to seal the open dentinal-tubules of the ultra-high polished dentin-surface (scale: 1 µm). (**h**) SEM image showing the dentinal tubule occlusion of microparticles in the absence of micro-brushing and air-blowing procedures.

Representative SEM images of dentin-surfaces treated with CHX-loaded/Ca(OH)_2_ microparticles carried on water (microparticles/carrier suspension), showed formation of an intact layer of uniformly disseminated spherical-shaped microparticles indicating successful attachment on the exposed dentin-surfaces (Figure 7b,c). Moreover, after the intentional removal of the overlying microparticles layer through micro-brushing and air-blowing, the ability of the microparticles to occlude the open dentinal-tubules of the ultra-high polished dentin-surface can be revealed Figure 7d–g. Even in the absence of such manipulative procedures, microparticles were seen associated to the orifice of the dentinal tubules Figure 7h. 

### 3.9. Biofilm Attachment/Confocal Microscopy Imaging

**Figure 8:**
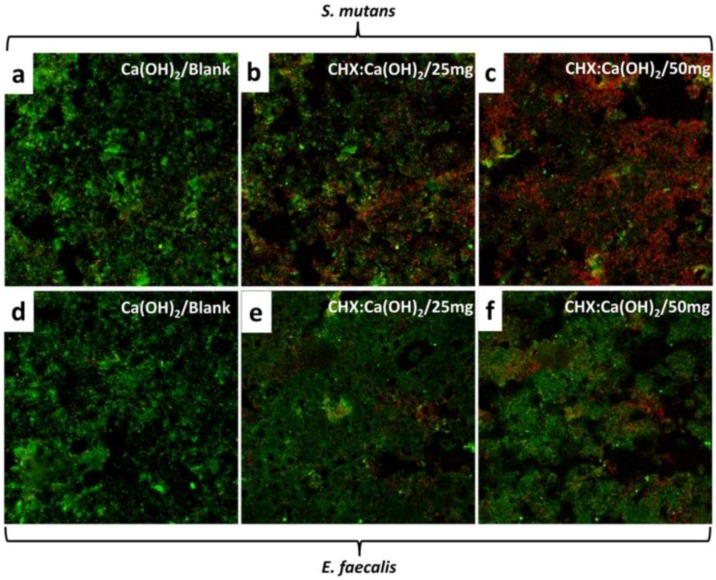
Selected confocal microscopy images showing Live/Dead assay of (**a**–**c**) *S. mutans* and (**d**–**f**) *E. faecalis* attached on dentin specimens treated with the Ca(OH)_2_/Blank, CHX:Ca(OH)_2_/25 mg and CHX:Ca(OH)_2_/50 mg microparticles, respectively at 3 days (scale: 15 µm). The distinction between live and dead bacterial cells could be noticed in the above images. (**a**,**d**) Ca(OH)_2_/Blank-treated dentin surfaces showed dense network of live bacterial cells covering the entire surface of treated dentin-surfaces with few dead bacteria in view. (**b**,**c**) The progressive antibacterial efficacy on *S. mutans* cells was evidenced from the increased red fluorescence contribution with dentin-specimens treated with increasing ratios of CHX-loaded Ca(OH)_2_ microparticles. (**e**,**f**) However, *E. faecalis* biofilms attached dentin-specimens treated with the CHX-loaded Ca(OH)_2_ microparticles revealed high coverage of predominant stubborn live cell fluorescence contribution as compared to the *S. mutans* biofilms.

Selected confocal microscopy images showing live/dead proportions of *S. mutans* and *E. faecalis* are shown in Figure 8. SYTO9-PI staining of biofilms revealed bacterial populations of living cells stained in green and dead cells with compromised cell membrane stained in red. An overlap of the live and dead bacteria produced a yellow to orange color at some locations. Biofilms attached on dentin-specimens treated with the Ca(OH)_2_/Blank microparticles showed heavy coverage with dense proportions of live bacteria with few dead bacteria in both bacterial strains (Figure 8a,d). The dentin specimens treated with the CHX-loaded/Ca(OH)_2_ microparticles demonstrated escalating effective antibacterial action on *S. mutans* biofilms denoted by the more observable contribution of dead bacteria with low fraction of live bacterial coverage (Figure 8b,c). For the *E. faecalis* biofilms (Figure 8e,f), attached to the microparticles treated dentin specimens, the fraction of predominant stubborn live cell fluorescence contribution become more expressed compared to the *S. mutans* biofilms.

### 3.10. In Vitro CHX-Release Profiles

**Figure 9:**
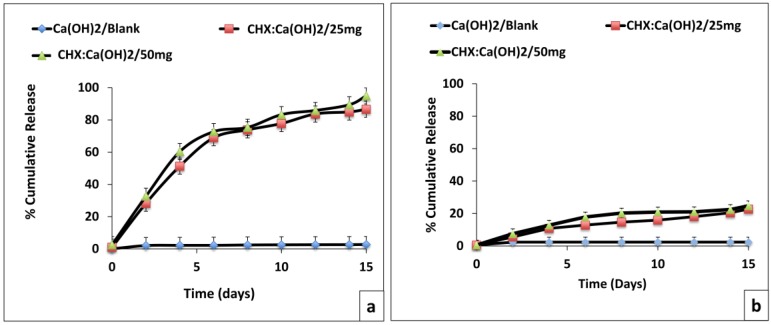
(**a**) The percentage (%) of cumulative CHX in vitro release from the CHX-loaded/Ca(OH)_2_ microparticles at 37 °C in phosphate buffered saline (PBS) at the physiological pH of 7.4 up to 15 days (in-vitro release-profile). (**b**) The percentage (%) of cumulative CHX ex vivo release from the CHX-loaded/Ca(OH)_2_ microparticles delivered to the dentin-substrate (ex-vivo release-profile) in phosphate buffered saline (PBS) at 37 °C at the physiological pH of 7.4 up to 15 days. **Note:** Statistical analysis of CHX-release was done with one-way ANOVA followed by Tukey-Kramer post-hoc test (*p* ≤ 0.05; significant). The Ca(OH)_2_/Blank microparticles were used as control.

From the in vitro CHX release experiment, an initial burst CHX-release was observed for ~1.5 days followed by a cumulative-release of 86.58% ± 2.1% and 94.94% ± 1.4% (at 15 days) for CHX-loaded/Ca(OH)_2_ microparticles at formulations of CHX:Ca(OH)_2_/25 mg and CHX:Ca(OH)_2_/50 mg, respectively (Figure 9a). The ex vivo CHX release-profiles from microparticles (at formulations of CHX:Ca(OH)_2_/25 mg and CHX:Ca(OH)_2_/50 mg) delivered to dentin-surfaces followed similar release-kinetics, with the highest cumulative value of 24.89% ± 9.5% from the CHX:Ca(OH)_2_/50 mg microparticles (Figure 9b).

## 4. Discussion

Despite significant advancement in the field of pulp therapy, the technique and philosophy of pulp capping remains a controversy [30]. Many clinicians have recommended the application of Ca(OH)_2_ for capping of accidentally injured human pulp [31]. With a longest record of clinical success, the alkalinity and ability to act as an effective pulp-protector still makes Ca(OH)_2_ a part of the practitioner’s armamentarium. Although treatment with Ca(OH)_2_ has been successful, dentistry has advanced further beyond the realm of traditional pulp-capping materials [32]. Researchers have exploited the properties of antiseptic compounds such as 2% chlorhexidine (CHX), a pulp-capping disinfectant and non-specific MMPs inhibitor, with an effort to amplify the antimicrobial properties of Ca(OH)_2_ and to achieve some beneficial synergism [33,34]. Although 2% CHX induced mild inflammatory pulp-response upon direct application, its combination with Ca(OH)_2_ not only eradicated microbes resistant to Ca(OH)_2_ alone, but also proved to be a successful intracanal medicament [35,36]. Accordingly, the synergistic effect of such medications may demand more complex interactions than simply the physical mixture of drugs. Moreover, the use of this combination has not yet been reported as a pulp-capping material. Therefore, in this work, we have formulated CHX-loaded/Ca(OH)_2_ microparticles, as a potential pulp-capping material, capable of providing more sustained release of CHX and thereby highlighting its property of substantivity. The main novelty of the current study lies in the present effort to immensely exploit Ca(OH)_2_ microparticles as a drug-delivery carrier. 

The CHX-loaded/Ca(OH)_2_ microparticles were synthesized by modified aqueous chemical-precipitation method (Figure 1a), owing to mild preparation conditions and feasibility in obtaining microparticles directly in suspension [27]. Microparticles with a unique morphology (Figure 3) were obtained, thereby offering a distinct advantage over other preparation-techniques [37]. Earlier, this simple inexpensive procedure was specifically adapted for fabricating nano-sized Ca(OH)_2_ [27,38,39]. To accommodate our need for a novel pulp-capping material, the procedure was improvised by reducing the amount of surfactant/triton X-100 with simultaneous drug (CHX) addition, targeting to synthesize microparticles with larger *z*-average diameters (Table 1). Moreover, CHX/Cl^−^ interaction resulting in precipitate formation and therefore, drug wastage has been minimized by addition of non-ionic surfactants [40]. With experiments primarily involving biological dentin-substrates, the inclusion of surfactant was strictly maintained at an absolute minimum (~0.15%). 

Chlorhexidine (CHX) has been well-recognized as a bisbiguanide, dicationic surfactant and a non-specific MMPs inhibitor [14]. Interestingly, CHX incorporation correlated with a significant reduction in *z*-average diameter from 5.3 ± 0.2 μm to 1.4 ± 0.3 μm with uniform size-distribution as indicated by DLS (Table 1). The intense association between -NH_2_ groups in CHX and the hydroxyl –OH groups present in Ca(OH)_2_ resulted in the formation of micrometric spheres of the CHX-loaded/Ca(OH)_2_, as verified by FTIR (Figure 4a). The initial surface retention of CHX due to competitive binding of triton X-100 [25] has attributed to surface alterations in the form of distinguishable “rounded-deposits” as identified by microscopic analyses (Figure 3b,c,f). Indication of structural changes upon entrapment was also confirmed by TGA findings (Figure 5a). Accordingly, the low ζ-potential of the Ca(OH)_2_/Blank exhibited a notable raise in the positive magnitude of the surface-charge (Table 1) suggesting successful CHX-uptake upon microparticle formation. The subsequent CHX-entrapment resulting in the uniform molecular dispersion of CHX in both formulations of CHX-loaded/Ca(OH)_2_ microparticles was apparent from XRD/DSC data (Figure 4b and Figure 5b). Although sufficient concentrations of the starting materials were used, with increasing CHX inclusion, less material would be left to bind CHX eventually leading to drug wastage. Moreover, the poor water solubility of CHX could have limited its entrapment to ~62.71% ± 1.2% as this procedure mainly involves water as the solvent (Table 1). As indicated in previous findings, the competitive binding of triton X-100 to CHX could have affected its incorporation in the carrier [41]. Being hydrophobic, the precipitation time of CHX is very short in water and to entrap it, the precipitation time of the particle carrier should be the same as the drug [42]. 

The defined spherical-shape, optimal *z*-average diameters (<2 μm), high alkalinity, moderate drug-entrapment of the CHX-loaded/Ca(OH)_2_ microparticles with sustained in vitro CHX-release kinetics (Table 1 and Figure 3b,c and Figure 9a,b) make them ideal drug delivery candidates. 

The dentin-substrates prepared by wet-grinding/ultra-high polishing and ultrasonic cleaning exposed the dentin-surface for treatment [43,44]. Microparticles applied on the prepared dentin-surfaces retained an intact dense layer of evenly-distributed spherical microparticles (Figure 7b,c) indicating successful delivery/attachment on the exposed dentin-surface. The unique ability of the positively-charged CHX- loaded/Ca(OH)_2_ microparticles to electrostatically bind to the negative-charges in trivalent phosphate in the hydroxyapatite crystals present in dentin could be addressed as the primary reason for successful attachment of microparticles on the dentin-surface [45]. The property of the microparticles to seal the dentinal-tubules is a key feature of an ideal pulp-capping agent. The method used for dentin-substrate preparation play a significant role in the attachment/uptake of microparticles. Moreover, the quality of the dentin-surface created by different techniques might be of importance in determining the degree of microparticle interaction that remains to be explored. 

Among the strategies for enhancing the uptake and even spreading of CHX-loaded/Ca(OH)_2_ microparticles on dentin-surfaces; the effect of micro-brush rubbing-action, the gentle air-blowing and water-blotting steps are arguably the most significant. The collective effect of these manipulative strategies might have assisted the microparticles to achieve significant degree of interaction with the dentin substrates. Additionally, further research should be done to clarify the penetration of microparticles into dentinal tubules.

The choice of distilled water has benefited as an experimental carrier/dispersion-phase for the CHX-loaded/Ca(OH)_2_ microparticles, considering the inert/less-soluble nature of both Ca(OH)_2_ and CHX in water [46]. Evidence from our pilot studies (unpublished observations) suggested that the microparticles/carrier ratio of 1/1 (*wt/v*) was the most effective in establishing a balance between sufficient microparticles retention onto dentin-surfaces and thus, not adversely affecting the functionality of microparticles following contact. In this study, dried microparticles were suspended in distilled water just prior to their application on dentin-substrates. Addressing such concerns, an immediate air-blowing and blot-drying steps were included after microparticles application, aiming to mitigate the debinding effect of water on CHX. Further research focusing on different types of microparticle carriers and microparticles/carrier ratios are currently under scrutiny. Moreover, the influence of microparticle storage related parameters, stability in dispersion-phase and their corresponding effect on microparticles delivery and CHX-release kinetics require further assessment.

The effects of Ca(OH)_2_ and CHX on the viability of the human mesenchymal stem cells (hMSCs) have been documented earlier [47,48]. The hMSCs population present in dental pulps are called dental pulp stem cells (DPSCs) [49]. Results of the current study showed enhanced biocompatibility of microparticles to hMSCs (after 24 h), at the proposed concentrations, in comparison to that of commercial Ca(OH)_2_ powder, pure CHX and Dycal, thereby demonstrating the prospects of a better and successful pulp-protective material (Figure 6b). It must be noted that for cytotoxicity experiments, dried powders of microparticles suspended in culture medium was used instead of the prepared microparticle suspensions. This type of dilution could influence and therefore, result in a change in the interaction upon initial exposure to the cells. Although, microparticles were delivered in the deepest layer of dentin adjoining the pulp, limitations pertaining to the thickness of dentin, patency of tubules and the effect of simulated pulpal pressure have to be further scrutinized. The presence of at least 0.5 to 1 mm dentin above the pulp and dentinal-fluid has known to render a buffering role that could further mitigate the toxic effects of microparticles, if any [50]. However, the tissue responses to calcium hydroxide are not always predictable [51]. Following pulp capping, a restoration must be applied. Therefore, toxicity of the pulp-capping material in addition to that of the restoration material must be considered [52]. It is noteworthy to mention that cytotoxicity assay only reflects the initial toxicity of microparticles utilized in this study. Further examination is essential to estimate the effect of sustained CHX-release for longer culture periods.

The antibacterial activity of pulp-capping agents has been widely evaluated with the agar-diffusion test [53,54]. Studies have attested to the diminished efficacy of CHX and Ca(OH)_2_ combination in eliminating the bacterial activity [55], as supported by current findings (Table 2). The antimicrobial activity of CHX is achieved with a pH range of 5.5 to 7.0 [56]. Alkalinizing the pH by addition of Ca(OH)_2_ deprotonates CHX and alters the molecular charge resulting in decreased solubility and reduced interaction with bacterial surfaces [57,58]. However, our data contradicted studies emphasizing on direct contact to achieve anti-bacterial effect [59]. Dycal and commercial Ca(OH)_2_ powder showed even lower antibacterial efficacy in accordance with previous studies [60]. The inherent resistance of bacterial biofilms to antimicrobial compounds has been well chronicled [61]. Within the limitations of our study, CHX-loaded/Ca(OH)_2_ microparticles showed more antibacterial efficiency on *S. mutans* (Figure 8b,c) as opposed to the recalcitrance exhibited by *E. faecalis* (Figure 8e,f) probably due to diffusion limitations posed by the extracellular matrix [62]. Nevertheless, these results can only be interpreted as a reference as they may not demonstrate the full clinical potential of the microparticles being tested. Future studies should be performed for longer time periods to further investigate the effect of prolonged CHX-release. An association between the release of OH^−^ ions and anti-microbial activity of Ca(OH)_2_ has been elucidated [63]. Further studies are needed to explore its effect on antibacterial action.

From the in vitro CHX-release profiles, we observed highest cumulative CHX-release from microparticles at the formulation of CHX:Ca(OH)_2_/50 mg at ~15 days (Figure 9a). The difference in CHX-release from different microparticles could be attributed to variations in drug-loading and strength of interactions involved in CHX-binding to the Ca(OH)_2_. The presence of CHX retained on the surface of Ca(OH)_2_ microparticles might contribute to rapid CHX-release (Figure 3e). The fact that Ca(OH)_2_ is slightly-soluble in water might have resulted in its escape from the dispersed phase to the aqueous release medium (PBS) with simultaneous liberation of CHX [64]. The hydrophilic affinity of polyethylene oxide head group present in triton X-100 could also have expedited the drug-release in vitro [65]. Moreover, the effect of composition of CaCl_2_ and NaOH used to form microparticles and their ionic dissociation into calcium (Ca^2+^) and hydroxyl (OH^−^) ions on the drug-release behavior has to be further probed.

The capacity of microparticles to remain attached to dentin-surfaces and seal the dentinal-tubules is vital for prolonged CHX-release following delivery. The ex vivo CHX-release profiles from microparticles delivered to dentin-surfaces followed the same pattern but has demonstrated lesser CHX-release than that determined in in vitro experiments (Figure 9b). Under such conditions, CHX after release from the microparticles could non-specifically bind to the mineral hydroxyapatite crystallites on the dentin-surface. The spaces between collagen-fibrils are occupied by mineral crystallites resulting in low porosity of the mineralized dentin [66]. Moreover, CHX released from microparticles occluding the dentinal-tubules could also diffuse and bind to the mineral inside the tubular wall. It is noteworthy that the surface-treated and sealed dentin-specimens were covered with filter-papers having pore-size smaller than the *z*-average diameter of the microparticles in order to prevent their escape to the surrounding PBS release medium from which CHX-release was detected. A low percentage (%) CHX-release in the surrounding PBS medium implied substantial CHX retention on the dentin-surface following release from CHX-loaded/Ca(OH)_2_ microparticles (Figure 9b). However, further evaluation is mandatory to directly quantify the amount of CHX retained on the dentin-surfaces.

Preservation of high alkalinity for prolonged time-periods has been regarded as one of the major advantages of Ca(OH)_2_ [67]. In the present study, Ca(OH)_2_ microparticles have been loaded with CHX, a component more functional at a lower pH environment. Nevertheless, it can be observed that CHX incorporation has not adversely affected the alkalinity of CHX-loaded/Ca(OH)_2_ microparticles as the high pH values were maintained throughout (Figure 6a), as indicated in earlier studies [18]. When performing pulp capping, such high alkalinity of Ca(OH)_2_ is known to incite an irritation potential, capable of inducing coagulation necrosis at the contact surface of the pulp [68]. Accordingly, the high pH values of the CHX-loaded/Ca(OH)_2_ microparticles (at 15 days) indicate their promising potential for reparative dentin barrier formation. However, experiments involving a vital pulp and corresponding histological evaluation of the dentin-bridge formation will provide fertile ground for future research. Previous literature findings have reported formation of mineralized tissue from ~7th to 10th day upon contact with Ca(OH)_2_, with complete bridge formation in 4 weeks sealing pulp exposure [68,69]. In the present study, in vitro CHX-release profiles show ~94.94% of CHX released in ~15 days (Figure 9a) and it is anticipated that the CHX-loaded/Ca(OH)_2_ microparticles continue to release CHX in a sustained manner beyond this specified time-period. Until the process of dentin bridge formation is complete, we expect that the CHX-released from the microparticles remain bound to dentin-matrix and protect the exposed pulp from bacterial microleakage and MMPs mediated degradation [70]. Moreover, the antibacterial effect of CHX-loaded/Ca(OH)_2_ microparticles is anticipated to minimize microleakage-mediated pulpal irritation. Furthermore, it would be essential to scrutinize whether the amount of CHX released from microparticles on dentin-substrates would be adequate for the long-term antibacterial efficacy and inhibition of endogenous host-derived dentin MMPs prior to establishing the next phase of this study.

## 5. Conclusions

This study introduces novel formulations of CHX-loaded/Ca(OH)_2_ microparticles with potential dental application as a pulp-capping material. The formulated CHX-loaded/Ca(OH)_2_ microparticles proved good antibacterial efficacy and demonstrated the ability to release significant quantities of CHX up to 15 days while maintaining high alkalinity. The potential ability of these drug-loaded microparticles to induce reparative dentin formation, non-specific MMPs inhibition and dentin-remineralization is promising and encourages further investigation.

## Figures and Tables

**Table 1:** Mean ± standard deviation of microparticles size (*z*-average), polydispersity index (PDI), percentage (%) encapsulation efficiency (EE), percentage (%) drug loading (DL) and percentage (%) microparticles recovery of the unloaded Ca(OH)_2_/Blank and CHX-loaded/Ca(OH)_2_ microparticles (at the formulations of CHX:Ca(OH)_2_/25 mg and CHX:Ca(OH)_2_/50 mg).

Formulations	*z*-Average Diameter (μm)	Zeta Potential (ζ) (mV)	Polydispersity Index (PDI)	Encapsulation Efficiency EE (%)	Drug Loading (DL) (%)	Microparticle Recovery (%)
Ca(OH)_2_/Blank	5.3 ± 0.2 ^A^	2.19 ± 0.4 ^A^	0.789 ± 0.038 ^A^	-	-	40.26 ± 2.6 ^A^
CHX:Ca(OH)_2_/25 mg	1.8 ± 0.5 ^B^	23.52 ± 4.5 ^B^	0.347 ± 0.010 ^B^	39.16 ± 1.6 ^A^	8.80 ± 6.1 ^A^	38.05 ± 3.5 ^A^
CHX:Ca(OH)_2_/50 mg	1.4 ± 0.3 ^B^	35.97 ± 8.6 ^C^	0.319 ± 0.093 ^B^	62.34 ± 2.4 ^B^	20.53 ± 3.4 ^B^	30.78 ± 1.9 ^B^

Groups with different superscript letters are statistically significant (*P* ≤ 0.05) within each column.Statistical analysis was done with one-way ANOVA followed by Tukey–Kramer post-hoc test.

**Table 2:** Inhibition zone diameters obtained from agar disk diffusion assay with *S. mutans* and *E. faecalis* in the presence of Dycal, the commercial Ca(OH)_2_ powder, the Ca(OH)_2_/Blank microparticles, the different formulations of CHX-loaded/Ca(OH)_2_ microparticles and the corresponding unencapsulated CHX (positive control).

Bacterial Strains Used	Diameters of Inhibition Zones (cm)* Measured at:						
	Ca(OH)_2_/Blank	CHX:Ca(OH)_2_/25 mg		CHX:Ca(OH)_2_/50 mg		Commercial Ca(OH)_2_ Powder	Dycal
		MP	CHX +ve Control	MP	CHX +ve Control		
*S. mutans*	0.72±0.05 ^A^	1.2±.0.37 ^B^	2.3±0.52 ^C^	1.8±0.28 ^D^	2.5±0.46 ^C^	0.62±0.18 ^AD^	0.48±0.15 ^D^
*E. faecalis*	0.49±0.13 ^A^	0.95±.0.24 ^B^	1.4±0.32 ^C^	1.1±0.31 ^B^	2.1±0.37 ^D^	0.46±0.17 ^A^	0.34±0.09 ^A^

Groups with different superscript letters are statistically significant (*P* ≤ 0.05) in each row. Statistical analysis was done with one-way ANOVA followed by Tukey–Kramer post-hoc test. MP: microparticles; CHX: Chlorhexidine.
